# The association between consecutive days’ heat wave and cardiovascular disease mortality in Beijing, China

**DOI:** 10.1186/s12889-017-4129-7

**Published:** 2017-02-23

**Authors:** Qian Yin, Jinfeng Wang

**Affiliations:** State Key Laboratory of Resources and Environmental Information System (LREIS), Institute of Geographic Sciences and Nature Resources Research, Chinese Academy of Sciences, A11, Datun Road, Beijing, 100101 China

**Keywords:** Heat wave, Cardiovascular disease, Consecutive days’ high temperature

## Abstract

**Background:**

Although many studies have examined the effects of heat waves on the excess mortality risk (ER) posed by cardiovascular disease (CVD), scant attention has been paid to the effects of various combinations of differing heat wave temperatures and durations. We investigated such effects in Beijing, a city of over 20 million residents.

**Methods:**

A generalized additive model (GAM) was used to analyze the ER of consecutive days’ exposure to extreme high temperatures.

**Results:**

A key finding was that when extremely high temperatures occur continuously, at varying temperature thresholds and durations, the adverse effects on CVD mortality vary significantly. The longer the heat wave lasts, the greater the mortality risk is. When the daily maximum temperature exceeded 35 °C from the fourth day onward, the ER attributed to consecutive days’ high temperature exposure saw an increase to about 10% (*p* < 0.05), and at the fifth day, the ER even reached 51%. For the thresholds of 32 °C, 33 °C, and 34 °C, from the fifth day onward, the ER also rose sharply (16, 29, and 31%, respectively; *p* < 0.05). In addition, extreme high temperatures appeared to contribute to a higher proportion of CVD deaths among elderly persons, females and outdoor workers. When the daily maximum temperature was higher than 33 °C from the tenth consecutive day onward, the ER of CVD death among these groups was 94, 104 and 149%, respectively (*p* < 0.05), which is considerably higher than the ER for the overall population (87%; *p* < 0.05).

**Conclusions:**

The results of this study may assist governments in setting standards for heat waves, creating more accurate heat alerts, and taking measures to prevent or reduce temperature-related deaths, especially against the backdrop of global warming.

**Electronic supplementary material:**

The online version of this article (doi:10.1186/s12889-017-4129-7) contains supplementary material, which is available to authorized users.

## Background

Global climate change has, of course, caused the climate to become warmer, but it has also increased the frequency, intensity, duration, and spatial extent of certain extreme weather events (such as heat waves and wind chill) [1–3]. Heat wave is a prolonged period of excessively hot weather. The precise definition of a heat wave varies between countries. The World Meteorological Organization (WMO) suggests that the key characteristics of a heat wave are the daily maximum temperature that is higher than 32 °C and duration of more than 3 days. In China, according to the climatic feature, the China Meteorological Administration states that the characteristics of a heat wave are daily maximum temperatures higher than or equal to 35 °C that last for more than 3 days [4]. From 1961 to 2010, the range of heat waves in China grew increasingly wide, they happened with ever greater frequency, and lasted exponentially longer [2]. In some areas, there were up to four heat waves in the summer, with durations of over 30 consecutive days [2].

The associations between heat waves and mortality have been well studied [5–8]. For example, several recent studies have reported increased risk of CVD mortality on heat-wave days as compared with non-heat-wave days; some of these studies have also reported that individual characteristics may help people to tolerate of prevent them from tolerating such extreme weather events [9–12]. In addition, several studies have researched the influence of heat waves based on different definitions (e.g. different temperature thresholds and durations) on CVD mortality [6, 13, 14]. Although several previous studies have investigated the CVD mortality risk posed by heat waves with different temperature thresholds and durations [15–17], almost all of them estimated the average increased risk of CVD mortality under different heat wave intensity and duration, but failed to distinguish that for different heat wave intensities and durations, the risks of CVD mortality could vary widely. For example, by analyzing the CVD mortality risk of heat waves on CVD in 43 U.S. cities (1987–2005), *Anderson* et al. (2011) found that the heat wave mortality risk increased by 2.49 and 0.38% for every 1 °F and 1 day increases in heat wave intensity and duration, respectively, in the United States. In the present study, we estimated the CVD mortality effects under various combinations of differing temperatures and durations combinations among a range of ages, sex and occupation groups, during the summers from 2010 to 2012 in Beijing. The aim was to enable the provision of a more accurate heat alert for the Chinese public.

## Methods

### Study area and meteorological data

Beijing, China’s capital, is a city of over 20 million residents that is characterized by a typical temperate monsoon climate: summers are hot and rainy.

We obtained the daily maximum temperature, daily mean relative humidity, and daily mean atmospheric pressure from 18 authorized meteorological observation stations in Beijing in the summers (1 June to 31 August) from 2010 to 2012; these values were taken from the public China Meteorological Data Sharing Service System website [18]. We estimated the mean values for daily maximum temperature, daily mean relative humidity, and daily mean atmospheric pressure across Beijing, using the Inverse Distance Weighting interpolation technique in ArcGIS 10.2 (Environmental Systems Research Institute, Redlands, CA, USA). For the same period, the daily PM_2.5_ concentrations at the US embassy station were obtained from the website of the Embassy of the United States in China. US embassy station is near the centre of the study region.

### Mortality data

Cardiovascular death cases among Beijing residents during the summers (1 June to 31 August) from 2010 to 2012 were obtained from the Chinese Center for Disease Control and Prevention (CDC). These cases were all identified by hospitals, and the death certificates recorded the following information: gender, date of death, age of death and occupation. According to the International Classification of Diseases (Version 10), CVD death cases were coded and classified into: I00-I99. All cases were classified into two groups, according to age: <65 years (younger) and ≥65 years (older). Based on the 75 occupations identified, the cases were divided into the following four categories: indoor workers, outdoor workers, unemployed, and unknown.

### Statistical methods

A generalized additive model (GAM) was used to analyze the excess mortality risk (ER) percentage of consecutive days’ exposure to high temperatures (see Eq. (1)). In this model, we also considered the effects of mean relative humidity, mean air pressure, daily temperature range (DRT) and air pollution [7, 8]:

1 $$ LogE\left[{Y}_t\right]=\alpha + C T{M}_t++ ns\left( DT{R}_t,\  d f\right)+ ns\left( R{H}_t, df\right)+ ns\left({P}_t, df\right)+ ns\left( P{M}_t, df\right)+ ns\left( Tim{e}_t, df\right)+\eta d o{w}_t $$

where *E(Y*_*t*_ *)* denotes the expected death number on day *t*, *α* is the intercept. The function *ns()* is a natural spline function. *RH*_*t*_ refers to the daily mean relative humidity (RH) on day *t*. The *df* (degree of freedom) for RH was 3. *DTR*_*t*_ refers to the daily temperature range(DTR) on day t, with *df* was 3. *PM*_*t*_ refers to the daily PM_2.5_ concentration on day t, with a *df* of 3. *P*_*t*_ refers to the daily mean air pressure on day *t*. The *df* for *P*_*t*_ was 3. The smooth term *Time*_*t*_ was used to control for secular trends [7]; the *df* of *time* was 7 in each year. *DOW*_*t*_ is the day of the week on day *t*, which is a categorical variable. *η* is a vector of coefficients. All of the *df* values for variables in this paper were chosen based on the Akaike Information Criterion (AIC) [19]. In contrast to other studies, we created a categorical variable, *CTM*, that refers to consecutive days of high temperature. A previous investigation had noted that the daily maximum temperature of minimum mortality risk in Beijing was 30.5 °C [7]. In this study, we chose the minimum mortality temperature (MMT, 30.5 °C) as the reference temperature and estimated the excess mortality risks of different temperature thresholds (32 °C, 33 °C, 34 °C, and 35 °C) and durations (1–11 days). For the various threshold values, we performed these calculations independently. The MMT was defined as the specific temperature associated to the lowest mortality risk. For example, if we chose 32 °C as the threshold, when the daily maximum temperatures were lower than or equal to 32 °C on a given day, the *CTM* value for this day was denoted as “REF” (reference). On the first day of exceeding the temperature threshold (32 °C), the *CTM* value was denoted as “Hot1” while on the second consecutive day, it was denoted as “Hot2” and so on (see the examples shown in Additional file 1: Table S1).

The ER was determined using the following formula. RR refers to the relative risk of CVD death at different temperature and durations, with the minimum mortality temperature (30.5 °C) used as the reference temperature:

2 $$ E R=\left( RR-1\right)*100\% $$

Because the estimation of relative risk in time series models may change obviously depending on parameter specifications [20–22], a sensitivity analysis was conducted by adjusting the *df* from 2 to 7 for DTR, daily mean air pressure, RH and PM_2.5_ concentration, and from 2 to 10 for each year for *time*. All of the *df* values for variables in this paper were chosen based on the Akaike Information Criterion (AIC) [19]. For different *df* value, the estimated values of relative risk varied slightly, but the trends were similar. For all statistical tests, *P* values <0.05 were considered statistically significant.

All statistical analyses were conducted using R statistical software (version 2.11.1; R Development Core Team 2010) and the mgcv package (Version 1.2.4).

## Results

### Statistical results

This study included 24,169 CVD death cases among Beijing residents in the summers (1 June to 31 August) from 2010 to 2012. Table 1 presents the daily mortality and meteorological data in the summers for Beijing over this period.

**Table 1:** Summary statistics for daily CVD deaths and meteorological variables in summer in Beijing, from 2010–2012

Category	Minimum	25%	Median	75%	Maximum	Mean ± SD
Daily meteorological data						
Maximum temperature (°C)	23.2	29.5	31.1	33	40.6	31.2±2.9
Mean relative humidity (%)	21	56.8	66	75	97	64.7±14.6
Mean air pressure (hPa)	990.4	998.1	1001	1004	1014	1001±4.6
Deaths by age						
≥65 years of age	39	62	71	79	144	71.9±12.9
0–64 years of age	6	13	15	18	36	15.7±4.3
Deaths by sex						
Male	24	38	44	50	88	44.4±7.5
Female	20	36	42	49	92	42.5±8.1
Deaths by occupation						
Outdoor	18	29	33	39	78	32.5±8
Indoor	17	33	38	44	67	37.6±7.1
Unemployed	2	7	9	12	19	9.5±4.3
Unknown	0	3	5	7	17	4.7±2.9

**Fig. 1:**
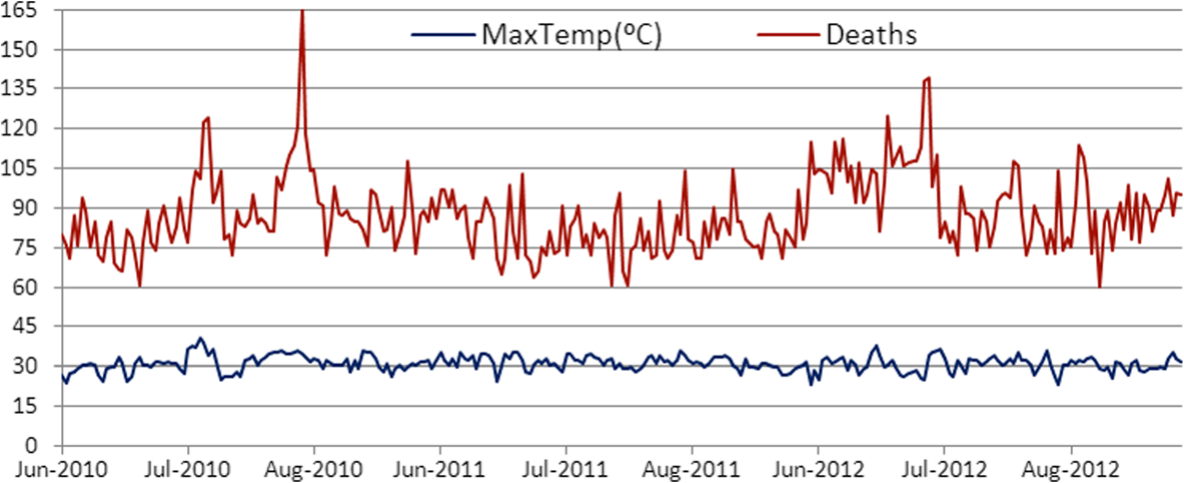
The daily maximum temperature and daily CVD deaths in summer from 2010 to 2012 in Beijing

Figure 1 presents the daily maximum temperatures and daily CVD deaths in the summers from 2010 to 2012 in Beijing. Table 2 shows the summary statistics for consecutive days on which residents were exposed to high temperatures; the temperature threshold was 32 °C. “Ref” denotes the days of daily maximum temperatures lower than or equal to 32 °C. Hot1, Hot2 … Hot11 stand for the consecutive days of daily maximum temperatures higher than 32 °C. The longest period in which there was a temperature over 32 °C lasted for 11 days, with an extreme maximum temperature of 40.6 °C.

**Table 2:** Consecutive days of exposed to high temperature (≥32 °C) during the summers from 2010 to 2012 in Beijing

	Ref	Hot1	Hot2	Hot3	Hot4	Hot5	Hot6	Hot7	Hot8	Hot9	Hot10	Hot11
Days	157	43	24	19	11	6	4	3	3	2	2	1

“Ref” denotes the days of daily maximum temperatures lower than or equal to 32 °C

### ER values of consecutive days’ high temperature

**Fig. 2:**
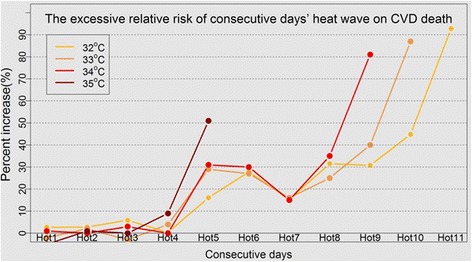
Percentage increase in CVD mortality due to consecutive days’ high temperatures for various threshold values, with an ER referential temperature of 30.5 °C

Figure 2 presents the ERs of CVD death in terms of high temperature over consecutive days (with thresholds of 32 °C, 33 °C, 34 °C, and 35 °C, respectively). The horizontal axis stands for the number of consecutive days with a maximum temperature higher than the threshold, while the vertical axis represents the ERs caused by various durations. Under these four conditions, the referential temperature of ER is 30.5 °C, which is the minimum mortality temperature in Beijing.

From Fig. 2, it can be observed that over the first 3 days, the ER of CVD death under the four conditions was generally consistent, being lower than 10% for all four. However, when daily maximum temperature was higher than 35 °C from the fourth day onward, the ER attributed to consecutive days’ extreme high temperature exposure underwent an increase to about 10 and 51% on the fourth and fifth days, respectively (*p* < 0.05). For the thresholds of 32 °C, 33 °C, and 34 °C, over the first 4 days, the ER of CVD death was generally low (under 10%), but it rose sharply when high temperatures continued to occur from the fifth day onward, increasing to 16, 29, and 31% as the days wore on (*p* < 0.05). On the seventh day, the ER has a clear harvesting effect (mortality displacement) [13, 17]. When the durations with daily maximum temperatures higher than 32 °C, 33 °C, and 34 °C reached 9, 10, and 11 days, respectively, the ERs of CVD death soared to 81, 87, and 93%, respectively (*p* < 0.05).

### ER values of the different subgroups

**Fig. 3:**
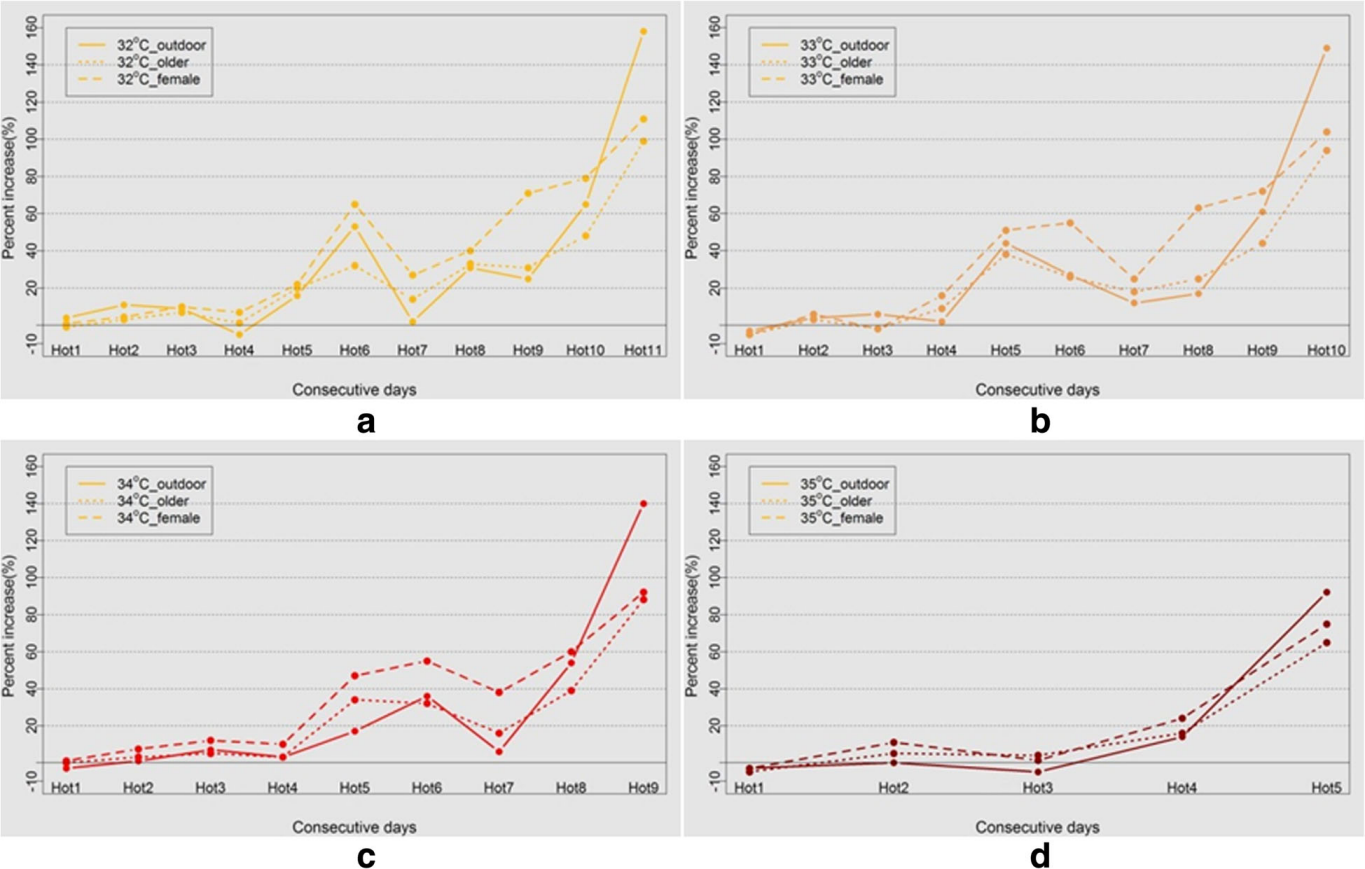
Percentage increases of CVD mortality in the various groups (female (dashed line), older (dotted line) and outdoor workers (solid line)) due to consecutive days’ high temperature, for varying threshold values: (**a**) 32 °C, (**b**) 33 °C, (**c**) 34 °C, and (**d**) 35 °C, with a reference temperature for ER of 30.5 °C

We estimated the percentages of ER increase, for the entire population as well as for various ages, both sexes and several occupation groups caused by high temperatures. Figure 3 presents the results. The horizontal axis represents the consecutive days of daily maximum temperature that was higher than the threshold, while the vertical axis signifies the ER caused by different durations of high temperature. Based on this analysis, we found that the effects of high temperature on CVD mortality among older people, female and outdoor workers were more serious than for the other groups. For example, when the daily maximum temperature was higher than 33 °C (see Fig. 3b) on the tenth consecutive day, the ERs of CVD death among elderly people, females and outdoor workers was 94, 104 and 149%, respectively, which is considerably higher than the ER for the total population (87%).

Tables 3 and 4 show the percentage increases and confidence interval (95% CI) for CVD mortality in the various groups listed in Figs. 2 and 3.

**Table 3:** Percentage increase (95% CI) in CVD mortality for the overall population, older people, females and outdoor workers

Percentage increase (95% CI)					
Category	Consecutive days	32 °C	33 °C	34 °C	35 °C
Overall population	Hot1	2.7(−2.1,7.7)	−2.4(−7.3,2.9)	1.1(−5.4,8.2)	−5(−11.1,1.6)
	Hot2	2.8(−3.2,9)	1.5(−5,8.5)	0.1(−7.6,8.5)	1.2(−7.5,10.6)
	Hot3	5.9(−0.4,12.6)	−3.2(−10.9,5.2)	2.7(−7.3,13.7)	0.3(−10.5,12.4)
	Hot4	0.4(−7.5,9)	4.4(−6.7,16.8)	−0.5(−13.3,14.2)	10.1(1.1,19.5)*
	Hot5	16.1(5.7,27.5)*	28.5(10.5,49.5)*	30.6(12.6,51.5)*	51(21.3,88.1)*
	Hot6	27.8(10.6,47.7)*	26.9(10.2,46.2)*	29.9(13.1,49.3)*	—
	Hot7	15.3(−1.2,34.6)	15.8(−0.4,34.7)	15(−0.8,33.4)	—
	Hot8	31.5(6.7,62.1)*	24.6(1.7,52.6)*	35(9.9,65.8)*	—
	Hot9	30.8(6.5,60.7)*	40(14.1,72.7)*	81(52,114.8)*	—
	Hot10	44.8(17.5,78.6)*	87(56.5,122.4)*	—	—
	Hot11	93(61.4,130.4)*	—	—	—
Older people	Hot1	−0.6(−5.8,4.8)	−4.2(−9.6,1.5)	0.2(−7,8)	−5.1(−11.9,2.3)
	Hot2	3.3(−3.2,10.3)	2.8(−4.4,10.6)	2.7(−6,12.1)	4.7(−5.1,15.4)
	Hot3	6.9(−0.1,14.3)	−1.7(−10.3,7.7)	4.7(−6.4,17)	4.4(−7.9,18.3)
	Hot4	1.2(−7.5,10.8)	8.5(−4,22.6)	3.2(−11.2,20.1)	15.6(−9.6,47.8)
	Hot5	19.5(7.9,32.3)*	37.7(17,62.2)*	33.9(13.8,57.7)*	65(30.4,109.4)*
	Hot6	32.3(13,54.8)*	26.3(8.1,47.6)*	32.1(13.4,53.7)*	—
	Hot7	13.7(−4.2,34.9)	18(0,39.2)*	16.1(−1.2,36.5)	—
	Hot8	33.3(5.9,67.8)*	25.2(0.2,56.3)*	39(11.1,74)*	—
	Hot9	30.7(4.3,63.8)*	44.5(15.2,81.2)*	88(56.2,126.9)*	—
	Hot10	47.8(17.6,85.9)*	94(60.2,134.3)*	—	—
	Hot11	99(63.9,140.9)*	—	—	—
Females	Hot1	1.1(−6.3,9)	−4.8(−12.4,3.5)	1.1(−9.1,12.5)	−3.3(−13,7.4)
	Hot2	4.4(−4.8,14.6)	5.9(−4.5,17.5)	7.5(−5,21.6)	10.9(−3.1,27)
	Hot3	9.6(−0.4,20.5)	−2.3(−14.2,11.2)	12.2(−4.1,31.1)	1.4(−15,21.1)
	Hot4	6.6(−6.2,21.2)	16(−2.3,37.7)	9.6(−11.1,35.2)	24.4(−11.6,75.2)
	Hot5	22.1(5.5,41.4)*	50.7(20.4,88.7)*	47(17.5,83.8)*	75(25.6,143.4)*
	Hot6	64.8(33.9,102.7)*	55.1(26.3,90.4)*	54.7(26.4,89.4)*	—
	Hot7	27(0.6,60.3)*	24.7(0.7,52.7)*	38.1(11.3,71.4)*	—
	Hot8	39.7(1.6,92)*	62.6(21.6,117.4)*	60.5(19.1,116.3) *	—
	Hot9	71(27.3,129.8) *	72.4(27.5,133) *	92(47.9,149.3) *	—
	Hot10	78.7(31.7,142.6)*	104(56.3,166)*	—	—
	Hot11	111(61,175.9)*	—	—	—
Outdoor workers	Hot1	3.5(−4.9,12.7)	−2.7(−11.3,6.7)	−2.8(−13.7,9.4)	−2.6(−12.9,8.9)
	Hot2	11(0.2,23)*	4.3(−7,17)	0.7(−12.3,15.8)	0.5(−13.6,16.8)
	Hot3	8.6(−2.4,20.7)	5.5(−8.3,21.5)	7.3(−9.8,27.7)	−5(−11,0)
	Hot4	−5.6(−18.6,9.6)	2.2(−16.3,24.7)	3(−18.6,30.5)	13.8(−21.9,65.8)
	Hot5	15.9(−1.7,36.6)	43.5(10.8,85.9)*	17.5(−10.7,54.6)	92(35.1,171.8)*
	Hot6	53(20.6,94.2)*	27.4(−0.8,63.7)	36(7,73)*	—
	Hot7	2.2(−23.6,36.8)	11.7(−15.2,47.1)	5.7(−19.2,38.3)	—
	Hot8	31.5(−10,92.2)	17.2(−18.6,68.8)	54(8.2,118.2)*	—
	Hot9	25.3(−13.4,81.4)	61(12.9,129.3)*	140(81.9,217.6)*	—
	Hot10	65(14.9,135.6)*	149(87.1,230.5)*	—	—
	Hot11	158(93.3,244.5)*	—	—	—

*: *p*<0.05; the referential temperature for ER is 30.5 °C

**Table 4:** Percentage increase (95% CI) in CVD mortality for younger people, males, indoor workers and the unemployed

Percentage increase (95% CI)					
Category	Consecutive days	32 °C	33 °C	34 °C	35 °C
Younger people	Hot1	13.4(−5.9,32.3)	5.5(−6.5,19.1)	4.3(−10.6,21.8)	−4.6(−18.3,11.5)
	Hot2	0.3(−13,15.6)	−4.1(−18,12.3)	−10.3(−25.9,8.5)	−13.3(−30.2,7.7)
	Hot3	0.6(−13.4,16.8)	−10.1(−26.5,9.9)	−7.2(−27.9,19.6)	−17.1(−37.4,9.8)
	Hot4	−3.5(−20.6,17.3)	−14.6(−36,13.9)	−16.4(−40.2,16.8)	−18.8(−54.2,44)
	Hot5	−0.6(−21.6,26.2)	−10.9(−40.4,33.3)	16.2(−19,66.6)	−6(−48.1,70)
	Hot6	5.9(−27.2,54)	29.2(−7.6,80.6)	19.3(−15.7,68.6)	—
	Hot7	22.6(−14.3,75.6)	5.8(−27,53.3)	9.5(−24.1,58.1)	—
	Hot8	23(−25.5,103.1)	21.2(−26.1,98.7)	15.8(−30.5,92.8)	—
	Hot9	30(−21.2,114.5)	20.6(−27.9,101.7)	43(−10.1,126.2)	—
	Hot10	29(−23.3,117)	49(−6.8,137.3)	—	—
	Hot11	59(−0.6,155.5)	—	—	—
Males	Hot1	−2.3(−9.3,5.3)	−3.6(−11.2,4.6)	−0.6(−10.4,10.4)	−6.9(−16.2,3.5)
	Hot2	2.2(−6.7,12.1)	−0.1(−9.9,10.8)	−1.9(−13.4,11.2)	−1.8(−14.9,13.2)
	Hot3	4.3(−5.1,14.6)	−1.1(−13,12.5)	−2.3(−16.8,14.7)	7.4(−10,28.1)
	Hot4	−3.8(−15.4,9.4)	1.7(−14.6,21.1)	−3(−22,20.7)	7(−24.8,52.4)
	Hot5	17.1(1.6,35)*	25.3(−1.3,59.1)	21.5(−4.3,54.3)	56(10.8,118.8)*
	Hot6	2.3(−19.7,30.5)	−0.9(−22.1,26.2)	9.9(−12.7,38.5)	—
	Hot7	0.7(−21.8,29.7)	11.3(−12.5,41.6)	−4.6(−25.4,22)	—
	Hot8	27.3(−8.7,77.7)	−7.8(−35,30.7)	17.5(−16.5,65.5)	—
	Hot9	−4.4(−32.8,36.1)	18(−16.4,66.6)	86(42,142.5)*	—
	Hot10	19.2(−15.9,68.9)	85(40.8,142.6)*	—	—
	Hot11	88(42.9,147.8)*	—	—	—
Indoor workers	Hot1	−2(−9.5,6.1)	−10.2(−17.8,-1.9)	−0.4(−11,11.5)	−8.9(−19.1,2.5)
	Hot2	−7.6(−16.5,2.1)	−6.8(−16.7,4.4)	−7(−18.9,6.6)	4.6(−10.3,22)
	Hot3	6.1(−4,17.3)	−12.1(−23.7,1.4)	2.1(−14.1,21.3)	21.1(0.2,46.3)*
	Hot4	−0.5(−13.1,13.8)	4.4(−13.1,25.5)	−12.9(−31.6,11)	2.5(−31.1,52.5)
	Hot5	28.2(10.5,48.8)*	22.9(−4.8,58.7)	46.6(15.6,85.8)*	42(−3.4,109.8)
	Hot6	12.1(−13.4,45)	27.4(0.8,61)*	26.5(−0.1,60.1)	—
	Hot7	30.6(2.5,66.3)*	20.8(−5.2,53.8)	32.3(5.1,66.7)*	—
	Hot8	20.5(−15.5,71.6)	30(−6.2,80)	13.1(−20.6,61.1)	—
	Hot9	36.6(−1.8,90.1)	15.6(−19.1,65.1)	61(20.6,115.9)*	—
	Hot10	20.7(−15.9,73)	65(22.3,121.1)*	—	—
	Hot11	70(26.4,129.8)*	—	—	—
Unemployed	Hot1	2.1(−11.9,18.3)	13.8(−2.9,33.5)	9.3(−11.1,34.5)	−2.6(−21.1,20.3)
	Hot2	14.6(−4.5,37.6)	34.6(6.9,69.5)*	37.1(12.6,66.8)*	19.3(−8.7,55.9)
	Hot3	10.2(−8.6,33)	6.3(−17.6,37)	2.7(−25.2,41.1)	−4.8(−33.2,35.7)
	Hot4	23.4(−3.2,57.3)	36.9(−1,89.2)	49.2(0.6,121.4)*	103(9.4,274.9)*
	Hot5	4.1(−23.3,41.2)	61(4,149.1)*	67.1(9.4,155.4)*	104(7,287)*
	Hot6	9.9(−29.1,70.3)	23.2(−19.2,87.6)	8.2(−29.8,66.8)	—
	Hot7	8.4(−31.8,72.5)	6.4(−33.9,71.4)	2.4(−36.1,64.1)	—
	Hot8	35.1(−28.1,153.9)	50.2(−19.5,180)	94(6.7,253.4)*	—
	Hot9	41(−24.9,164.9)	109(14.5,282.3)*	103(18.5,248)*	—
	Hot10	103(10.3,274.1)*	116(24.9,272.6)*	—	—
	Hot11	108(19.8,261.3)*	—	—	—

*: *p*<0.05; the referential temperature for ER is 30.5 °C

Based on the above results, we offer an innovative proposal for a heat alert (see Table 5), which considers both the high temperature threshold and duration.

**Table 5:** A new proposal for a heat alert for Beijing residents

	Threshold	Duration
Alert1	32 °C	>4 consecutive days
Alert2	33 °C	>4 consecutive days
Alert3	34 °C	>4 consecutive days
Alert4	35 °C	>3 consecutive days

## Discussion

Although many studies have examined the effects of heat wave on the excess mortality risk of CVD, almost all of them estimated the average increased risk of CVD mortality under different heat wave intensity and duration, but failed to distinguish that for the different intensity and durations, the risks of CVD death can vary widely. In the present study, we estimated the CVD mortality effects under various combinations of temperature and duration combinations. A key finding was that when high temperatures occur continuously, at varying temperature thresholds and different durations, the adverse effects on CVD mortality vary significantly. The longer the heat wave lasts, the greater the mortality risk is. For example, when the daily maximum temperature higher than 35 °C occurred continuously, on the fourth and fifth days, the ERs attributed to consecutive days’ high temperature exposure underwent an obvious increase to about 10 and 51% (*p* < 0.05); and for the thresholds of 32 °C, 33 °C, and 34 °C, on the fifth day, the ERs also rose sharply to 16, 29, and 31%, respectively (*p* < 0.05). Because very few studies have specifically focused on the various combinations of temperature threshold and duration of heat wave on CVD mortality. So it is difficult to compare our results with those of previous studies, However, a few studies have reported that given different temperature thresholds and durations, heat waves have adverse impacts on non-accidental and coronary heart disease (CHD) mortality [16, 17]. *Sheridan* et al. (2014) found that all non-accidental mortality risk increased by 21.2% on the fifth day of heat wave in New York. By analyzing the CHD mortality risk caused by heat waves in Beijing (2000–2011), *Tian* et al. (2013) found that CHD mortality risk increased by about 60% on the seventh days of heat wave. Another reason why we could not compare our results with these of previous studies is that the increased risk of CVD mortality they estimated for heat-wave days is compared with non-heat-wave days. Whereas, in the present study, we choose the minimum-mortality temperature (MMT) as the reference temperature for relative mortality risk. In my opinion, using MMT as the reference temperature is more reasonable.

Based on these results, we set forward a new proposal for a heat alert, which considers both the high temperature threshold and duration. When the duration of daily maximum temperatures higher than 35 °C reaches to 4 days or the duration with daily maximum temperatures higher than 32 °C, 33 °C or 34 °C reach 5 days, governments should both issue heat alerts, medical institutions and emergency centers should prepare greater quantities of medical resources, such as intravenous fluids, oxygen, and extra beds for rapid treatment [23]. The CVD-vulnerable population should take certain actions to prevent the adverse effects of high temperature, such as using air conditioning, avoiding outdoor activities, wearing light clothing, drinking more water, and so on [24, 25]. Whereas there was only one heat alert standard before, daily maximum temperatures higher than 35 °C for a duration exceeding 3 days.

Our results confirm that exposure to high temperatures can increase CVD mortality. This result is consistent with certain extant studies [26, 27]. Several underlying mechanisms have been offered as an explanation of the risk posed by high temperature in terms of CVD mortality. One argument is that at low and moderate temperatures, body heat is transferred to the environment, essentially through a process of convection. However, at high temperatures, the body can only regulate its normal temperature through sweat and evaporation [28]. These processes can be related to hemoconcentration and may induce a failure of thermoregulation. In addition, high temperatures may lead to an increase in blood viscosity and cardiac output which may result in dehydration, hypotension, surface blood circulation increases and even the impairment of peripheral vascular endothelial function [29, 30]. This may place additional pressure on those with existing health problems, including cardiovascular diseases.

In this study, we found that the adverse effects of heat waves among elderly persons and females are more serious than for younger people and males. This result is consistent with previous studies [5]. The higher vulnerability levels observed among the elderly and female may be due to their different physiopathological responses to heat stress [31, 32], as well as variations in social and living conditions [33, 34]. When expose to high temperatures, elderly persons’ ability to regulate their body temperature is weaker than that of the young. Accordingly, high temperatures could have more serious effects on them. Consecutive days’ high temperature exposure had more serious effects on outdoor workers’ CVD mortality rates than those found for the other groups. These results show that longer periods of high temperature exposure increase the risks of CVD mortality. However until now, few countries have implemented occupational heat exposure regulations and created standards for outdoor heat exposure [35],

This study has several limitations. Firstly, CVD mortality is determined not only by weather conditions and an individual’s age, sex and occupation, but also by socioeconomic factors, which differ between cities. Therefore, these other variables should be taken into account when comparing results from different areas. Secondly, in our study, we used the meteorological data taken from observation stations instead of data based on personal exposure. This may cause some estimation error. Lastly, due to data inaccessibility, we only analyzed the effect of heat wave on total CVD death cases, and did not consider more detailed causes of CVD.

## Conclusion

In this individual case-based study, we found that increases in CVD mortality rates appear to be associated with high temperature, especially when these occurring over consecutive days.

The results of this study may assist governments in setting standards for heat waves, creating more accurate heat alerts, and taking measures to prevent or reduce temperature-related deaths, especially against the backdrop of global warming.

## Additional file

Additional file 1: Table S1. Categorical variable: CTM (consecutive days of high temperature). Threshold is 32 °C. (DOCX 34 kb)

## Abbreviations

AIC: Akaike information criterion
CDC: Chinese center for disease control and prevention
CI: Confidence intervals
CVD: Cardiovascular disease
DTR: Daily temperature range
ER: Excess mortality risk
GAM: Generalized additive model
MMT: Minimum-mortality temperature
RH: Relative humidity
RR: Relative risk
WMO: World meteorological organization
