# Supplementary material for: The association between consecutive days’ heat wave and cardiovascular disease mortality in Beijing, China
Source: BMC Public Health. 2017 Feb 23;17:223. doi: 10.1186/s12889-017-4129-7 (PMC5322604; doi:10.1186/s12889-017-4129-7)
Supplement: Additional file 1: Table S1. — Categorical variable: CTM (consecutive days of high temperature). Threshold is 32 °C. (DOCX 34 kb) [file 12889_2017_4129_MOESM1_ESM.docx]

- **Table S1.** Categorical variable: CTM (consecutive days of high temperature). Threshold is 32^o^C

| **Date** | **Daily maximum temperature (^o^C)** | **CTM** |
| --- | --- | --- |
| 2010-7-1 | 27.6 | REF |
| 2010-7-2 | 36.7 | Hot1 |
| 2010-7-3 | 37.8 | Hot2 |
| 2010-7-4 | 36.9 | Hot3 |
| 2010-7-5 | 40.6 | Hot4 |
| 2010-7-6 | 39.2 | Hot5 |
| 2010-7-7 | 34.3 | Hot6 |
| 2010-7-8 | 36.5 | Hot7 |
| 2010-7-9 | 24.8 | REF |
| 2010-7-10 | 26.5 | REF |
| 2010-7-11 | 33.5 | Hot1 |
| 2010-7-12 | 34.2 | Hot2 |
| … | … | … |
